# Antimicrobial resistance of commensal *Enterococcus faecalis* and *Enterococcus faecium* from food-producing animals in Russia

**DOI:** 10.14202/vetworld.2022.611-621

**Published:** 2022-03-18

**Authors:** Dmitry A. Makarov, Olga E. Ivanova, Anastasia V. Pomazkova, Maria A. Egoreva, Olga V. Prasolova, Sergey V. Lenev, Maria A. Gergel, Nataliya K. Bukova, Sergey Yu Karabanov

**Affiliations:** 1 Department of Food and Feed Safety, Federal State Budgetary Institution, The Russian State Center for Animal Feed and Drug Standardization and Quality (VGNKI), Moscow, Russia; 2 Department of Biotechnology, Federal State Budgetary Institution, The Russian State Center for Animal Feed and Drug Standardization and Quality (VGNKI), Moscow, Russia; 3 Department of Immunobiological Drugs, Federal State Budgetary Institution, The Russian State Center for Animal Feed and Drug Standardization and Quality (VGNKI), Moscow, Russia; 4 Testing Centre, Federal State Budgetary Institution The Russian State Center for Animal Feed and Drug Standardization and Quality (VGNKI), Moscow, Russia; 5 Department of Experimental Clinic and Research Laboratory for Bioactive Substances of Animal Origin, V. M. Gorbatov Federal Research Center for Food Systems, Moscow, Russia

**Keywords:** animals, antimicrobial resistance, enterococci, *Enterococcus faecium*, *Enterococcus faecalis*, livestock

## Abstract

**Background and Aim:**

Although *Enterococcus faecalis* and *Enterococcus* *faecium* are common members of human and animal gut microbiota, their resistance to different antimicrobials makes them important pathogens. Multidrug-resistant enterococci often contaminate foods of animal origin at slaughterhouses. The World Health Organization and the World Organization for Animal Health recommend including animal-derived enterococci in antimicrobial resistance (AMR) monitoring programs. This study aimed to fill a literature gap by determining the current AMR prevalence of *E. faecalis* and *E. faecium* from different food-producing animals in Russia.

**Materials and Methods:**

Samples of biomaterial were taken from chickens (n=187), cattle (n=155), pigs (n=49), turkeys (n=34), sheep (n=31), and ducks (n=31) raised at 28 farms in 15 regions of Russia. Isolates of *E. faecalis* (n=277) and of *E. faecium* (n=210) (487 isolates in total; 1 isolate per sample) were tested for resistance to 12 antimicrobials from 11 classes using the broth microdilution method. Three criteria were used for the interpretation of minimum inhibitory concentration: Epidemiological cutoff values (ECOFFs) from the European Committee on Antimicrobial Susceptibility Testing (EUCAST) and Clinical and Laboratory Standards Institute (CLSI) clinical breakpoints. The AMR cloud online platform was used for data processing and statistical analysis.

**Results:**

A difference of >10% was found between *E. faecalis* and *E. faecium* resistance to several antimicrobials (erythromycin, gentamycin, tetracycline, chloramphenicol, ciprofloxacin, and streptomycin). In total, resistance to most antimicrobials for enterococci isolates of both species taken from turkeys, chicken, and pigs was higher than cattle, sheep, and ducks. The highest levels were found for turkeys and the lowest for ducks. Among antimicrobials, resistance to bacitracin and virginiamycin was 88-100% in nearly all cases. High levels of clinical resistance were found for both bacteria species: Rifampicin (44-84%) from all animals, tetracycline (45-100%) from poultry and pigs, and erythromycin (60-100%), ciprofloxacin (23-100%), and trimethoprim-sulfamethoxazole (33-53%) from chickens, turkeys, and pigs. No vancomycin-resistant isolates were found. Most isolates were simultaneously resistant to one–three classes of antimicrobials, and they were rarely resistant to more than three antimicrobials or sensitive to all classes.

**Conclusion:**

Differences in resistance between enterococci from different farm animals indicate that antimicrobial application is among the crucial factors determining the level of resistance. Conversely, resistance to rifampicin, erythromycin, tetracycline, and ciprofloxacin found in enterococci from farm animals in our study was notably also found in enterococci from wild animals and birds. Our results may be partly explained by the intrinsic resistance of *E. faecium* and *E. faecalis* to some antimicrobials, such as trimethoprim/sulfamethoxazole and bacitracin.

## Introduction

Enterococci are Gram-positive facultative anaerobes that live as commensals in the gastrointestinal tract of various organisms, including humans, other mammals, birds, reptiles, and insects; they are also found in soil, water, and food [1,2]. Conversely, enterococci are opportunistic human pathogens which have commonly become resistant to many antimicrobial agents. Typically harmless for healthy individuals, in immunocompromised persons, enterococci can cause endocarditis, bacteremia, infections of the urinary tract, wounds, and the intra-abdominal and pelvic areas, and superinfections [3,4]. Antimicrobial application may lead to the development of resistance in the enterococci population. Horizontal gene transfer is an important factor in accelerating resistance spread [5].

Enterococci are second to staphylococci as the leading cause of nosocomial infections, with a mortality rate of up to 23% [6]. The genus *Enterococcus* consists of over 50 species, and *Enterococcus faecalis* and *Enterococcus faecium* account for more than 80% of human clinical isolates [7], being among the most common in the gut microbiota of humans and animals [2].

At slaughterhouses, fecal enterococci can contaminate food products of animal origin. Some studies reported that over 90% of food samples of animal origin are contaminated with enterococci, mostly with *E. faecalis*, followed by *E. faecium* [8,9]. These two species were shown to be present as contaminants in raw and processed food such as cheese, fish, sausages, minced beef, pork, and ready-to-eat foods [10]. *E. faecalis* and *E. faecium* have also been associated with infections in food animals and poultry [11-13].

Correlation between the use of antimicrobial agents in each country and the occurrence of associated resistance in *E. faecalis* and *E. faecium* from animals was observed in several European countries, including resistance to the growth promoters bacitracin and virginiamycin [14,15]. A study by Ghosh and Zurek [14] demonstrated that enterococci of food-animal origin, particularly vancomycin-resistant strains, can colonize the human digestive tract.

The World Organization for Animal Health (OIE) recommends the surveillance of antimicrobial resistance (AMR) of *E. faecium* and *E. faecalis* isolates from animals [16]. The World Health Organization (WHO) recommends including *Enterococcus* spp. in programs of foodborne AMR monitoring [17]. Enterococci from animals are a part of the National AMR Monitoring System in the United States [18]. A number of studies exist on animal-commensal enterococci from other countries [7].

Data on AMR of enterococci from food animals in the Russian Federation are scarce. Animal isolates are not included in systematic monitoring programs. This study aimed to fill a literature gap by determining the current AMR prevalence of *E. faecalis* and *E. faecium* from different food-producing animals in Russia.

## Materials and Methods

### Ethical approval

The study was approved by the Federal Service for Veterinary and Phytosanitary Surveillance (Rosselkhoznadzor, AAAA-A20-1200127900477).

### Study period, location, and sample collection

The samples of biomaterial (n=487) were collected from March 2017 to December 2021 from 28 farms located in 15 regions of Russia. Most of the farms were located in central Russia (n=15); others were located in the Kaliningrad region (n=1), the Caucasus (n=1), Siberia (n=3), the Volga region (n=4), and the Urals (n=3). The samples were collected from the biomaterial of animals that appeared healthy: poultry (chickens, turkeys, and ducks), cattle, pigs, and sheep. The number of isolates (277 of *E. faecalis* and 210 of *E. faecium* in total), depending on animal species and age category, farms, bacteria species, and type of biomaterial are shown in Table-1. Only one isolate was taken from each sample (*E. faecalis* or *E. faecium*).

**Table-1:** The number of samples depending of farm, sample type, and age group.

Animal species and age groups	*Enterococcus* species	Number of samples, Sample type, (Age group), Farm №.
Chicken (*Gallus gallus domesticus*): Broiler chicken (Br), hatchlings (Ha) n=187	*Enterococcus faecalis*	38CS (Br) - Farm №5
		2FE (Br) - Farm №6
		18FE (Ha) - Farm №7
		24CS (Br) - Farm №8
		4FE (Br) - Farm №9
		5CS (Br) - Farm №10
		7FE (Br) - Farm №12
		13CS (Br), 15FE (Br) - Farm №18
		20CS (Br) - Farm №23
	*Enterococcus faecium*	5CS (Br) - Farm №5
		5CS (Br) - Farm №8
		4FE (Br) - Farm №9
		6FE (Br) - Farm №12 6CS (Br) - Farm №13
		18CS (Br) - Farm №18
Turkey (*Meleagris gallopavo*): Adult (Ad), Turkey poult (Tp) n=34	*Enterococcus faecium*	11LI (Ad), 3 CS (Ad) - Farm №3
		20CS (Tp) - Farm №4
Duck (*Anas platyrhynchos domesticus*): Adult n=31	*Enterococcus faecalis*	9CS, 7FE, 4 LI - Farm №15
		7FE, 4LI - Farm №20
Cattle (*Bos taurus*): Adult n=155	*Enterococcus faecalis*	9FE, 7LI- Farm №2
		2LI - Farm №16
		5VS - Farm №19
		6FE, 2LI - Farm №24
		3LI - Farm №25
		9FE, 1VS- Farm №27
		4LI, 3FE – Farm № 30
	*Enterococcus faecium*	1FE, 1LI - Farm №16
		21FE- Farm №17
		10VS, 5FE- Farm №19
		6FE, 5LI - Farm №24
		8FE, 2LI - Farm №25
		7FE, 7VS- Farm №27
		15FE, 10VS – Farm № 28
		4FD, 3FE, 3LI – Farm № 30
Pig (*Sus scrofa domesticus*): Feedlot swine (Fs)/Young (Yo): 1-2 y.o./Piglet (Pi):<1 y.o. n=49	*Enterococcus faecalis*	5FE (2 Pi, 3 Fs), 7LI (4Ad, 3Pi) - Farm №1
		25FE (13 Fs, 6 Pi, 6 Yo) - Farm №11
		10 FE (8Pi, 2Fs), 2LI (Ad) - Farm №26
Sheep (*Ovis aries*): Adult n=31	*Enterococcus faecium*	

Totally 487 isolates (277 of *Enterococcus faecalis* and 210 of *Enterococcus faecium*), Sample types: CS=Carcass swab, FE=Feces, LI=Litter, VS=Vagina swab. Farm № 1 is located in Altai Krai, №2=In Bashkortostan, №3-9=In Belgorod oblast, №10-11=In Chelyabinsk oblast, № 12-14=In Dagestan, №15=In Kaliningrad oblast, №16-17=In Kaluga oblast, №18-19=In Novosibirsk oblast, №20-22=In Orenburg oblast, №23=In Penza oblast, №24-25=In Ryazan oblast, №26=In Sverdlovsk oblast, №27=In Tver oblast, №28=In Voronezh oblast, №29-30=In Yaroslavl oblast

### Bacteria isolation and identification

Bacteria were isolated using chromogenic broth as a liquid enrichment medium (HiCrome enterococci broth, HiMedia, India). Slanetz-Bartley agar was used to obtain individual colonies of enterococci (Slanetz-Bartley agar HiMedia). The samples were incubated at 37°C for 24 h on both media. Identification was performed on a time-of-flight mass spectrometer (MALDI Biotyper Microflex system, Bruker, Germany). The identification results were confirmed by the API^®^ Strep biochemical kit (BioMerieux, France).

### Antimicrobial susceptibility testing (AST)

The following 12 antimicrobial standards were purchased from Sigma-Aldrich, USA: Ampicillin, bacitracin, chloramphenicol, ciprofloxacin, erythromycin, gentamicin, rifampicin, streptomycin, tetracycline, trimethoprim, vancomycin, and virginiamycin. AST was performed by broth microdilution according to the Clinical and Laboratory Standards Institute (CLSI) standard [19].

The following strains were used for quality control: *E. faecalis* ATCC 29212 and *Escherichia coli* ATCC 25922. The quality control ranges for *E. faecalis* ATCC 29212 were as follows: ampicillin (2-0.5 μg/mL), chloramphenicol (16-4 μg/mL), ciprofloxacin (2-0.25 μg/mL), erythromycin 4-1 μg/mL), gentamicin 16-4 μg/mL), rifampicin (4-0.5 μg/mL), tetracycline (32-8 μg/mL), trimethoprim (0.5-0.12 μg/mL), and vancomycin (4-1 μg/mL).

The quality control ranges for *E. coli* ATCC 25922 were as follows: Ampicillin (8-2 μg/mL), chloramphenicol (8-2 μg/mL), ciprofloxacin (0.016-0.004 μg/mL), gentamicin (1-0.25 μg/mL), rifampicin (16-4 μg/mL), tetracycline (2-0.5 μg/mL), trimethoprim (2-0.5 μg/mL), and trimethoprim in combination with sulfamethoxazole (concentration ratio1:19): <0.5 and 9.5 μg/mL, respectively.

## Interpretation of minimum inhibitory concentration (MICs) and analysis of the results

MICs were interpreted according to three systems of criteria: (1) For microbiological resistance, the European Committee on AST (EUCAST) epidemiological cutoff values (EUCAST ECOFFs) [20], (2) for clinical resistance, the EUCAST clinical breakpoints v.2021 (EUCAST 2021) [21], and (3) the CLSI clinical breakpoints v.2021 [22]. Data were interpreted according to these three systems to allow other researchers to compare and combine their results with ours, regardless of their research criteria. Comparing and combining data allows for the assessment of general trends and makes possible general conclusions in the AMR distribution between different countries, animals, humans, and the environment the basis for the One Health approach elaborated on and promoted by the WHO, OIE, and Food and Agriculture Organization [17].

Intermediately-resistant isolates were treated as sensitive for CLSI criteria to account for high levels of resistance found in our study and in response to the fact that EUCAST revised the “intermediate” category to the new “susceptible, increased exposure” category, thereby raising the inevitability of the application of higher concentrations of antimicrobials.

To determine clinical multidrug resistance, we used data on eight antimicrobials belonging to eight different classes; penicillins (ampicillin), phenicols (chloramphenicol), quinolones (ciprofloxacin), aminoglycosides (gentamicin), ansamycins (rifampicin), glycopeptides (vancomycin), macrolides (erythromycin), and tetracyclines (tetracycline).

### Statistical analysis

Analyses of the results were carried out using a free-access AMR cloud online platform (https://amrcloud.net), which was developed by the Institute of Antimicrobial Chemotherapy (Smolensk, Russia) [23]. The principles of data analysis are similar to the internationally-recognized AMR map online database, and they are described in the corresponding publication [24]. AMR cloud processes the data on MICs for different antimicrobials and isolates, which were uploaded by the user in an Excel spreadsheet. AMR cloud interprets data according to three systems of criteria, and it allows data to be presented depending on user needs in graph, table, MIC distribution, and map form. Statistical evaluation was done automatically, allowing results to be presented with the 95% confidence interval. The AMR cloud tool saves time and allows for deep, comprehensive analysis, and clear visualization of the data on AMR.

The web platform is developed using the R programming language and software environment for statistical computing. It makes use of the following packages: “shiny” for an interactive web interface, “ggplot2” for graphics, “data table” and “DT” for aggregating data in tables, “visNetwork” for network visualization, “leaflet” for geographical maps, and “highcharter” for wrapping Highcharts JavaScript graphics library and modules [24].

## Results and Discussion

*E. faecalis* and *E. faecium* microbiological resistance patterns with MICs interpreted against the EUCAST ECOFFs [20] are shown in Table-2. Clinical resistance patterns with MICs interpreted against EUCAST2021 [21] and CLSI 2021 [22] clinical breakpoints are shown in Tables-2 and 3, respectively. *E. faecalis* resistance was tested in isolates from cattle, chickens, ducks, and pigs; likewise, *E. faecium* resistance was tested from cattle, chickens, turkeys, and sheep.

**Table-2:** Resistance patterns of enterococci isolates, depending on the animal species (interpretation against ECOFFs).

Antimicrobial	*Enterococcus faecalis*			*Enterococcus faecium*		
	
	Number of isolates tested	Resistant isolates, %	95% Confidence interval	Number of isolates tested	Resistant isolates, %	95% Confidence interval
	**Cattle**			**Cattle**		
AMP	51	0	0-7	104	1	0.2-5.3
BAC	34	100	89.9-100	27	96.3	81.7-99.3
CHL	51	9.8	4.3-21	104	0	0-3.6
CIP	51	0	0-7	104	1	0.2-5.3
ERY	51	13.7	6.8-25.7	104	35.6	27-45.1
GEN	51	0	0-7	104	0	0-3.6
STR	51	2	0.4-10.3	104	0	0-3.6
TET	51	15.7	8.2-28	104	7.7	4-14.5
VAN	51	0	0-7	104	0	0-3.6
VIR	34	32.4	19.1-49.2	27	100	87.5-100
	**Chicken**			**Chicken**		
AMP	146	0	0-2.6	41	14.6	6.9-28.4
CHL	146	21.9	16-29.3	41	0	0-8.6
CIP	146	24.7	18.4-32.2	41	39	25.7-54.3
ERY	146	60.3	52.2-67.9	41	61	45.7-74.3
GEN	146	1.4	0.4-4.9	41	19.5	10.2-34
STR	146	3.4	1.5-7.8	41	7.3	2.5-19.4
TET	146	95.2	90.4-97.7	41	48.8	34.3-63.5
VAN	146	0	0-2.6	41	0	0-8.6
	**Duck**			**Turkey**		
AMP	31	0	0-11	34	5.9	1.6-19.1
CHL	31	3.2	0.6-16.2	34	0	0-10.2
CIP	31	3.2	0.6-16.2	34	100	89.9-100
ERY	31	9.7	3.4-24.9	34	100	89.9-100
GEN	31	3.2	0.6-16.2	34	94.1	80.9-98.4
STR	31	3.2	0.6-16.2	34	76.5	60-87.6
TET	31	45.2	29.2-62.2	34	100	89.9-100
VAN	31	0	0-11	34	0	0-10.2
	**Pig**			**Sheep**		
AMP	49	0	0-7.3	31	0	0-11
BAC	24	87.5	69-95.7	6	100	61-100
CHL	49	22.5	13-35.9	31	0	0-11
CIP	49	20.4	11.5-33.6	31	0	0-11
ERY	49	87.8	75.8-94.3	31	16.1	7.1-32.6
GEN	49	53.1	39.4-66.3	31	0	0-11
STR	49	30.6	19.5-44.5	31	0	0-11
TET	49	100	92.7-100	31	19.4	9.2-36.3
VAN	49	0	0-7.3	31	0	0-11
VIR	24	100	86.2-100	6	100	61-100

AMP = Ampicillin, BAC = Bacitracin, CHL = Chloramphenicol, CIP = Ciprofloxacin, ERY = Eythromycin, GEN = Gentamicin, RIF = Rifampicin, STR = Streptomycin, TET = Tetracycline, TRI = Trimetohoprim, TRI-SUL = Trimethoprim-Sulfamethoxazole, VAN = Vancomycin, VIR = Virginiamycin

**Table-3:** Resistance patterns of enterococci isolates depending on the animal species (interpretation against EUCAST 2021).

Antimicrobial	*Enterococcus faecalis*		*Enterococcus faecium*	
	
	Resistant isolates, %	95% Confidence interval	Resistant isolates, %	95% Confidence interval
	**Cattle (n=51)**		**Cattle (n=104)**	
AMP	0	0-7	1	0.2-5.3
CIP	0	0-7	1	0.2-5.3
GEN	0	0-7	0	0-3.6
STR	2	0.4-10.3	0	0-3.6
TRI	11.8	5.5-23.4	14.4	8.9-22.4
TRI-SUL	11.8	5.5-23.4	0	0-3.6
VAN	0	0-7	0	0-3.6
	**Chicken (n=146)**		**Chicken (n=41)**	
AMP	0	0-2.6	14.6	6.9-28.4
CIP	24.7	18.4-32.2	39	25.7-54.3
GEN	1.4	0.4-4.9	4.9	1.4-16.1
STR	3.4	1.5-7.8	4.9	1.4-16.1
TRI	20.6	14.8-27.8	34.2	21.6-49.5
TRI-SUL	32.9	25.8-40.9	36.6	23.6-51.9
VAN	0	0-2.6	0	0-8.6
	**Duck (n=31)**		**Turkey (n=34)**	
AMP	0	0-11	5.9	1.6-19.1
CIP	3.2	0.6-16.2	100	89.9-100
GEN	3.2	0.6-16.2	94.1	80.9-98.4
STR	3.2	0.6-16.2	52.9	36.7-68.6
TRI	3.2	0.6-16.2	52.9	36.7-68.6
TRI-SUL	3.2	0.6-16.2	52.9	36.7-68.6
VAN	0	0-11	0	0-10.2
	**Pig (n=49)**		**Sheep (n=31)**	
AMP	0	0-7.3	0	0-11
CIP	20.4	11.5-33.6	3.2	0.6-16.2
GEN	51	37.5-64.4	0	0-11
STR	30.6	19.5-44.5	0	0-11
TRI	55.1	41.3-68.2	0	0-11
TRI-SUL	53.1	39.4-66.3	0	0-11
VAN	0	0-7.3	0	0-11

AMP=Ampicillin, BAC=Bacitracin, CHL=Chloramphenicol, CIP=Ciprofloxacin, ERY=erythromycin, GEN=gentamicin, RIF=Rifampicin, STR=Streptomycin, TET=Tetracycline, TRI=Trimetohoprim, TRI-SUL=Trimethoprim-Sulfamethoxazole, VAN=Vancomycin, VIR=Virginiamycin

### Microbiological resistance

We found notable differences (>10% of resistant isolates) between *E. faecalis* and *E. faecium* for several antimicrobials: (1) Erythromycin and virginiamycin for cattle; and (2) tetracycline, gentamicin, ciprofloxacin, chloramphenicol, and ampicillin for chicken.

As shown in Table-2, the microbiological resistance of *E. faecalis* to most antimicrobials was observed in the following descending order: pig→chicken→cattle→duck. More than 20% resistance to all antimicrobials in pig isolates was found, except for ampicillin and vancomycin (0% for both bacteria). In contrast, for ducks, the percentage of R-isolates was <10% for all antimicrobials, except for tetracycline (45%). Resistance in *E. faecium* was observed in the following descending order: turkey→chicken→cattle→sheep. Our results correlate with the trend that antimicrobials are used in higher amounts for chickens and pigs than cattle [25].

Low resistance for isolates in ducks is likely explained by the fact that in Russia, duck meat is consumed in negligible quantities compared with that of cattle, pigs, and chickens, and duck farms typically exhibit a low density of birds, possibly leading to lower antimicrobial use. Our group previously demonstrated that for *E. coli*, the AMR of isolates from turkeys was the highest and that from chickens and pigs, the resistance was higher than from cattle, which is consistent with data from the present study [26].

### Resistance to bacitracin and virginiamycin

The highest levels of microbiological resistance were found for virginiamycin and bacitracin, two antimicrobials used as feed additives to promote growth. The percentages of R-isolates from cattle, pigs, and sheep were equal to or nearly 100% for both antimicrobials. In Russia, no antimicrobials are authorized for growth promotion, but bacitracin and virginiamycin are approved for use in farm animals for treatment and prophylactic purposes. Studies indicated higher use of bacitracin and virginiamycin in feed than other antimicrobials [27]. Thus, using bacitracin and virginiamycin as feed additives is likely the key factor determining the high levels of resistance found in our study. However, data on the intrinsic resistance of enterococci to bacitracin should be considered [28].

It should be noted from the methodological point of view that quality control was performed without using corresponding concentration ranges since we did not find them in either the CLSI or EUCAST standards. However, no notable shifts of concentrations were found compared with the EUCAST standard distributions [29] (data not shown).

### Clinical resistance

A notable difference in clinical resistance (>10% of resistant isolates) was discovered between *E. faecalis* and *E. faecium* for ciprofloxacin, erythromycin, rifampicin, and trimethoprim-sulfamethoxazole from cattle, and ampicillin, ciprofloxacin, chloramphenicol, tetracycline, trimethoprim, and trimethoprim-sulfamethoxazole from chickens using EUCAST2021 [21] and CLSI 2021 [22] criteria (Tables-3 and 4).

**Table-4:** Resistance patterns of enterococci isolates depending on the animal species (interpretation against CLSI 2021).

Antimicrobial	*Enterococcus faecalis*		*Enterococcus faecium*	
	
	Resistant isolates, %	95% Confidence interval	Resistant isolates, %	95% Confidence interval
	**Cattle (n=51)**		**Cattle (n=104)**	
AMP	0	0-7	1	0.2-5.3
CHL	9.8	4.3-21	1	0.2-5.3
CIP	2	0.4-10.3	20.2	13.6-28.9
ERY	13.7	6.8-25.7	35.6	27-45.1
GEN	0	0-7	0	0-3.6
RIF	84.3	72-91.8	70.2	60.8-78.1
STR	2	0.4-10.3	0	0-3.6
TET	15.7	8.2-28	7.7	4-14.5
VAN	0	0-7	0	0-3.6
	**Chicken (n=146)**		**Chicken (n=41)**	
AMP	0	0-2.6	14.6	6.9-28.4
CHL	21.9	16-29.3	4.9	1.4-16.1
CIP	24.7	18.4-32.2	75.6	60.7-86.2
ERY	60.3	52.2-67.9	61	45.7-74.3
GEN	0	0-2.6	0	0-8.6
RIF	48	40-56	43.9	29.9-59
STR	3.4	1.5-7.8	4.9	1.4-16.1
TET	95.2	90.4-97.7	48.8	34.3-63.5
VAN	0	0-2.6	0	0-8.6
	**Duck (n=31)**		**Turkey (n=40)**	
AMP	0	0-11	5	1.4-16.5
CHL	6.5	1.8-20.7	50	35.2-64.8
CIP	6.5	1.8-20.7	100	91.2-100
ERY	9.7	3.4-24.9	100	91.2-100
GEN	0	0-11	0	0-8.8
RIF	83.9	67.4-92.9	50	35.2-64.8
STR	3.2	0.6-16.2	45	30.7-60.2
TET	45.2	29.2-62.2	100	91.2-100
VAN	0	0-11	0	0-8.8
	**Pig (n=49)**		**Sheep (n=31)**	
AMP	0	0-7.3	0	0-11
CHL	22.5	13-35.9	0	0-11
CIP	22.5	13-35.9	32.3	18.6-49.9
ERY	87.8	75.8-94.3	16.1	7.1-32.6
GEN	0	0-7.3	0	0-11
RIF	65.3	51.3-77.1	77.4	60.2-88.6
STR	30.6	19.5-44.5	0	0-11
TET	100	92.7-100	19.4	9.2-36.3
VAN	0	0-7.3	0	0-11

AMP=Ampicillin, BAC=Bacitracin, CHL=Chloramphenicol, CIP=Ciprofloxacin, ERY=erythromycin, GEN=gentamicin, RIF=Rifampicin, STR=Streptomycin, TET=Tetracycline, TRI=Trimetohoprim, TRI-SUL=Trimethoprim-Sulfamethoxazole, VAN=Vancomycin, VIR=Virginiamycin

Comparing data on resistance of isolates from different animals (using EUCAST2021and CLSI 2021 criteria), the same trend was found as for microbiological resistance: In general, for most antimicrobials, the percentages of R-isolates of *E. faecalis* from chickens and pigs seem to be greater than those from cattle and ducks. The percentages of R-isolates of *E. faecium* from turkeys and chickens are generally greater than from cattle and sheep.

Among all bacteria/animal combinations, the overall resistance of *E. faecium* isolates from turkeys seems to be the greatest: >50% for six out of seven antimicrobials tested (according to EUCAST2021); >45% for six out of seven antimicrobials (according to CLSI 2021), including 100% resistance to ciprofloxacin, tetracycline, and erythromycin (CLSI 2021).

Previously, we demonstrated similar resistance orders to most antimicrobials for commensal *E. coli* from turkeys, chicken, cattle, and pigs [26]. Thus, the high levels of AMR of enterococci and *E. coli* isolated from turkeys is likely not due to the nature of the bacteria but rather to the higher antimicrobial usage for turkeys in Russia. Further research is needed to correlate antimicrobials use in Russia and commensal enterococci resistance. All turkey isolates were from two farms belonging to an agricultural cluster with intensive farming practices in Belgorod Oblast.

Resistance to streptomycin and gentamicin was similar for most bacteria/animal combinations. However, for *E. faecalis* from pigs and *E. faecium* from turkeys, more isolates were resistant to gentamicin than streptomycin (51% vs. 31% for pigs and 94% vs. 53% for turkeys), suggesting a different genetic mechanism of resistance to these two different members of the aminoglycoside family.

### Resistance to trimethoprim and sulfamethoxazole

**Figure-1:**
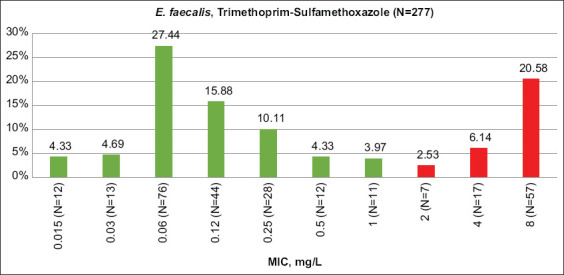
Trimethoprim-sulfamethoxazole minimum inhibitory concentration distribution among *Enterococcus faecalis* isolates. Green columns – percent of susceptible isolates, red – resistant (EUCAST 2021 breakpoints), N – number of isolates.

**Figure-2:**
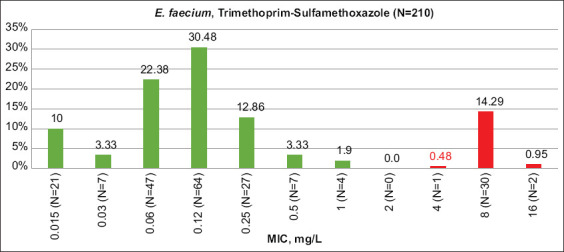
Trimethoprim-sulfamethoxazole minimum inhibitory concentration distribution among *Enterococcus faecium* isolates. Green columns – percent of susceptible isolates, red – resistant (EUCAST 2021 breakpoints), N – number of isolates.

The highest absolute percentages of R-isolates for both bacteria species from all tested animal species were shown for trimethoprim and the combination of trimethoprim and sulfamethoxazole (EUCAST2021) (Table-3). Resistance to other antimicrobials was higher than for trimethoprim and its combination with sulfamethoxazole in only *E. faecium* from turkeys and sheep. In some cases, resistance to the combination of drugs was even higher than trimethoprim alone. After accounting for quality control, this case is difficult to explain. High levels of resistance to trimethoprim and sulfamethoxazole were previously shown by our group for *E. coli* from different farm animals [26]. Trimethoprim and sulfamethoxazole are not among the most often-applied antimicrobials in animal husbandry. According to the recent OIE report for 103 countries, including Russia [29], the reported quantities of sulfonamides (including trimethoprim) was only 5.4%, compared with 34% and 11% of tetracyclines and penicillins, respectively. Considering that in Russia alone, antimicrobial consumption is consistent with the OIE trend (data not shown), and the difference in resistance between trimethoprim-sulfamethoxazole and tetracycline or penicillin is likely not due to differing levels of antimicrobial use but rather to the important role of active horizontal transfer of resistance-conferring genes in both enterococci and *E. coli*. Literature also indicates high intrinsic resistance of enterococci to trimethoprim-sulfamethoxazole because enterococci absorb folic acid from the environment, thereby bypassing the inhibiting effect of trimethoprim-sulfamethoxazole on folate synthesis [30]. Figures-1 and 2 show the MIC distribution of the trimethoprim-sulfamethoxazole combination.

Furthermore, we found high resistance levels to rifampicin, tetracyclines, ciprofloxacin, and erythromycin (Tables-2 and 3), whereas resistance to ampicillin, streptomycin, and chloramphenicol was lower in most cases. For example, among *E. faecium* isolates from chickens, 44%, 76%, 61%, and 49% were resistant to rifampicin, ciprofloxacin, erythromycin, and tetracycline, respectively, whereas only 15%, 5%, and 0% were resistant to ampicillin, chloramphenicol, and gentamycin/vancomycin, respectively (Table-3).

### Resistance to vancomycin

Resistance to vancomycin is the most important issue for enterococci. In the WHO global priority pathogens list, vancomycin-resistant *E. faecium* is categorized as a high priority for urgently needing new antimicrobials [31]. We did not find any single vancomycin-resistant isolate following microbiological and clinical breakpoints.

### Resistance to rifampicin

**Figure-3:**
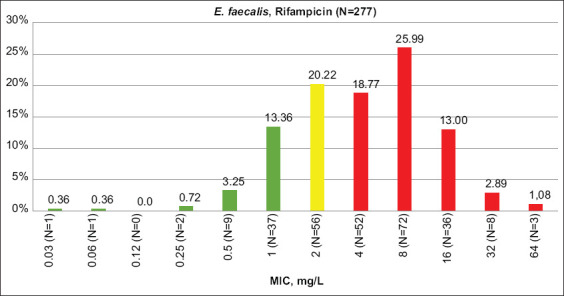
Rifampicin minimum inhibitory concentration distribution among *Enterococcus faecalis* isolates. Green columns – percent of susceptible isolates, yellow – intermediate resistant, red – resistant (CLSI 2021 breakpoints), N – number of isolates.

**Figure-4:**
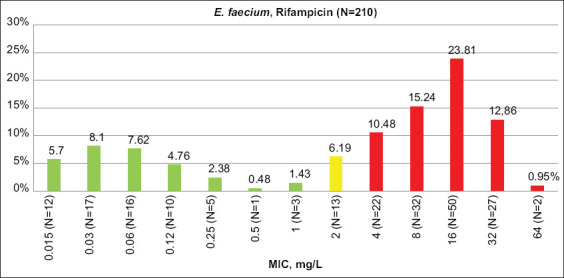
Rifampicin minimum inhibitory concentration distribution among *Enterococcus faecium* isolates. Green columns – percent of susceptible isolates, yellow – intermediate resistant, red – resistant (CLSI 2021 breakpoints), N – number of isolates.

According to CLSI criteria, resistance to rifampicin (no <40%) was the highest among all antimicrobials tested. Resistance to this drug was lower for enterococci from turkeys and chickens than cattle and sheep, showing this trend to be opposite that of other antimicrobials. Rifampicin MIC distribution is shown in Figures-3 and 4.

### Comparison with other studies

Our results partly correlate with the data on enterococci isolated from animals in other countries. The studies discussed below were performed using the CLSI clinical breakpoints [22]. For enterococci isolated from pigs and poultry in one China Province, the authors report frequent resistance (50-93%) to tetracycline, erythromycin, and rifampicin, an intermediate resistance rate (~30%) to phenicols (chloramphenicol and florfenicol) and enrofloxacin, and a low resistance rate to penicillins (4% to ampicillin) [32]. In *E. faecalis* isolated from chickens in Colombia, high resistance was found for tetracycline (98.1%), erythromycin (82.0%), and enrofloxacin (80.6%) [33].

Wild animals were shown to be the source of enterococci, with significant resistance levels. Enterococci from Brazilian wild pampas foxes and Geoffroy’s cats (mostly belonging to *E. faecalis* and *E. faecium* species) were found to be resistant to rifampicin (94%), erythromycin (72%), ciprofloxacin/norfloxacin (40%), streptomycin (38%), and tetracycline (26%) [34]. It is notable that, in our study, resistance to streptomycin in wild animal isolates was higher than in farm animal isolates, except for turkeys. *E. faecium* isolates from wild birds caught in the Azores archipelago were reportedly resistant to ciprofloxacin (33%), tetracycline (45%), erythromycin (15%), and ampicillin (5%) [35].

These data indicate the important role of natural mechanisms in the development of enterococci resistance to antimicrobial agents. However, in the abovementioned studies on wild animals, sampling was performed in places that are not free from animal husbandry activities and anthropogenic load. Thus, the influence of antimicrobials consumed by animals and people cannot be excluded.

### Multiple resistances

**Figure-5:**
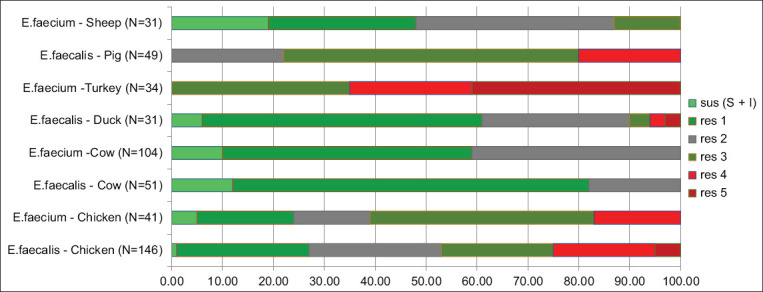
Patterns of simultaneous resistance to different classes of antimicrobials. Sus (S+I) means the percent of isolates susceptible to all antimicrobials tested (susceptible + intermediate categories according to CLSI 2021). Res 1 means the percent of isolates resistant to at least 1 antimicrobial, Res 2 – to 2 antimicrobials, etc.

Patterns of multiple resistances of both bacteria species from different animal species according to CLSI criteria [22] are presented in Figure-5. Susceptible and intermediate categories of resistance were merged.

The percentage of isolates susceptible to all classes of antimicrobials according to CLSI 2021 [22] exceeds 10% only for *E. faecium* from sheep and *E. faecalis* from cattle. No multi-resistant (i.e., simultaneously resistant to three or more classes of antimicrobials) isolates were found in bacteria from cattle, whereas in enterococci from other animals, the percentage of multi-resistant isolates varies from 0 to 10% (*E. faecalis* from ducks) to 100% (*E. faecium* from turkeys).

Most of the isolates were simultaneously resistant to one–three antimicrobial classes, and they were determined primarily by resistance to rifampicin, erythromycin, tetracycline, and ciprofloxacin. Simultaneous resistance to five classes was detected only for isolates from poultry: >60% of isolates from turkeys (*E. faecium*) and < 10% from ducks (*E. faecalis*) and chickens (*E. faecalis*). No isolates resistant to six or more classes of antimicrobials were found, indicating the availability of therapeutic options in case of infection caused by strains of enterococci with the discovered profiles of resistance.

## Conclusion

Here, we demonstrate that AMR of *E. faecalis* and *E. faecium* from chickens, turkeys, and pigs appear to be higher than those from cattle, sheep, and ducks, and resistance was highest among bacteria from turkeys. Given the same trend was previously shown for commensal *E. coli*, the difference in intensity of antimicrobial use for different animal species is likely to be among the crucial factors determining the levels of commensal enterococci resistance. The highest levels of microbiological resistance were found for bacitracin and virginiamycin (close to 100% in most cases). High levels of clinical resistance were demonstrated to tetracycline, erythromycin, ciprofloxacin, and the trimethoprim-sulfamethoxazole combination for both bacteria from turkeys, chickens, and pigs. In contrast, resistance to rifampicin was high regardless of the animal species. Rifampicin resistance was even higher in cattle and sheep than in turkeys and poultry. The intrinsic resistance properties of *E. faecium* and *E. faecalis* may explain the demonstrated prevalence of resistance for some antimicrobials, for example, trimethoprim-sulfamethoxazole and bacitracin. According to the literature, high resistance levels similar to our data were discovered for rifampicin, erythromycin, tetracycline, and ciprofloxacin from animals in other countries. This was found in wild animals and birds as well as in food-producing animals, highlighting the importance of investigating natural reservoirs of resistance and their transfer between farms and the environment. The analysis of simultaneous resistance showed that most of the *E. faecalis* and *E. faecium* isolates were resistant only to one or two classes of antimicrobials, suggesting sufficient therapeutic options exist in case of infection by strains of the same resistance pattern. However, of concern are the high levels of resistance to rifampicin and other drugs for *E. faecalis* and *E. faecium*. This matter and limiting antimicrobials use for farm animals should be considered to develop national programs of AMR surveillance. Further research is needed to investigate genes of resistance and factors determining the resistance prevalence.

## Authors’ Contributions

DAM: Data analysis and drafted the manuscript. OEI: Designed the experiment. AVP, MAE, SVL, NKB: Sampling, isolation, and antimicrobial susceptibility testing. OVP: Preparation of the manuscript. MAG: Supervision of the study. SYK: Antimicrobial susceptibility testing and preparation of the manuscript. All authors have read and approved the final manuscript.
